# The Underappreciated Benefits of Interleaving for Category Learning

**DOI:** 10.3390/jintelligence11080153

**Published:** 2023-08-02

**Authors:** Lan Anh Do, Ayanna K. Thomas

**Affiliations:** Department of Psychology, Tufts University, Medford, MA 02155, USA; ayanna.thomas@tufts.edu

**Keywords:** category learning, interleaving effect, feature descriptions, study schedule

## Abstract

The present study examined the effects of study schedule (interleaving vs. blocking) and feature descriptions on category learning and metacognitive predictions of learning. Across three experiments, participants studied exemplars from different rock categories and later had to classify novel exemplars. Rule-based and information-based categorization was also manipulated by selecting rock sub-categories for which the optimal strategy was the one that aligned with the extraction of a simple rule, or the one that required integration of information that may be difficult to describe verbally. We observed consistent benefits of interleaving over blocking on rock classification, which generalized to both rule-based (Experiment 1) and information-integration learning (Experiments 1–3). However, providing feature descriptions enhanced classification accuracy only when the stated features were diagnostic of category membership, indicating that their benefits were limited to rule-based learning (Experiment 1) and did not generalize to information-integration learning (Experiments 1–3). Furthermore, our examination of participants’ metacognitive predictions demonstrated that participants were not aware of the benefits of interleaving on category learning. Additionally, providing feature descriptions led to higher predictions of categorization even when no significant benefits on actual performance were exhibited.

## 1. Introduction

Category induction happens when one learns to acquire the recurring pattern that defines a concept or a category membership through studying their exemplars (Ashby and O’Brien 2005; Carvalho and Goldstone 2015b; Noh et al. 2014). Inductive category learning is an important skill, as it allows people to generalize their knowledge attained from a limited amount of experience to a wider range of novel exemplars beyond the original learning event (Ashby and O’Brien 2005). The importance of category induction has been emphasized in various fields of study, such as art (Kornell and Bjork 2008), geology (Whitehead et al. 2021), ornithology (Birnbaum et al. 2013; Wahlheim et al. 2011), medical diagnoses (Chen et al. 2015; Hatala et al. 2003) and mathematics (Taylor and Rohrer 2010; Rohrer et al. 2015). Much research, therefore, has focused on identifying the effective learning techniques to promote category induction.

Research demonstrates that category induction can be executed via different learning systems depending on the category structure (Ashby et al. 1998; Ashby and Maddox 2005, 2011; Ashby and O’Brien 2005). Rule-based category learning takes place when the category characteristics are easy to verbalize. Such a learning task is optimized by a hypothesis testing learning system in which learners generate and test verbalizable rules. By contrast, there are other categories that are not as easy to verbally define. Learners should integrate information from numerous examples of a certain category to capture the category membership. This process is thus referred to as information-integration category learning. Learning information-integration categories requires a more holistic, procedural-based learning system in which students should implicitly associate the examples with a recurring category-level pattern.

The present research examines how different kinds of study schedules and the inclusion of descriptive information promote category learning for categories that are inclined more to rule-based category learning or more to information-integration categorization processes. Here, “study schedule” refers to the way that exemplars are sequenced during a study session. In a blocked schedule, the exemplars are grouped by category, whereas in an interleaved schedule, the exemplars are randomly intermixed across categories. The study schedule that optimizes learning depends on the nature of study materials (Carvalho and Goldstone 2015a, 2019; Noh et al. 2016) and the learning processes through which students learn the categories (Noh et al. 2016).

A great deal of research has focused on the effects of study schedules on category induction (see Kang 2016 for an overview); that is, the participants learned different categories by studying a series of exemplars but were not explicitly informed about the rules or the characteristic features that defined category membership. Instead, they had to induce the pattern by themselves (Birnbaum et al. 2013; Kornell and Bjork 2008; Wahlheim et al. 2011). The present study extends the literature by examining how interleaving and blocking schedules may affect category learning differently when students are explicitly provided with verbal explanations that describe the important features of a category. We sought to examine how these two study schedules may interact with feature descriptions when participants learned to categorize different rock types. Feature descriptions are an integral part of rock category learning and are often included in textbooks (e.g., Marshak 2019; Tarbuck and Lutgens 2018) and laboratory manuals (e.g., Cronin 2018). Research also shows that feature descriptions can enhance rock identification and classification (Meagher et al. 2022; Miyatsu et al. 2019). Therefore, the present study aimed to identify the learning techniques that can optimize different category learning processes by combining different study schedules with feature descriptions for rock category learning.

The final goal of the present study was to investigate whether learners are aware of the factors that result in effective category learning. Metacognition is important to examine because the way that participants judge their current learning can affect subsequent study behaviors such as the amount of time that they allocate for studying and the specific items they select to study (Efklides 2014; Metcalfe 2009). Research has demonstrated that learners often suffer from various metacognitive biases, as they judge their learning status based on undiagnostic cues such as the ease or difficulty of task processing (e.g., Reber and Greifeneder 2017). Learners are also likely to misinterpret higher effort as an indicator of poor learning (Kirk-Johnson et al. 2019; Onan et al. 2022). This can result in a preference for ineffective study methods that generate a higher sense of fluency and require less effort, such as a blocking schedule rather than interleaving (Kirk-Johnson et al. 2019; Onan et al. 2022; also see de Bruin et al. 2023 for a review). The present investigation extends previous works by examining the impact of different study schedules combined with feature descriptions on metacognitive predictions about category learning. Specifically, we investigated how study schedules and the presence or absence of feature descriptions affected participants’ metacognitive predictions of their future test performance.

## 2. Literature Review

### 2.1. Research on Study Schedule

Research suggests that blocking and interleaving schedules promote learning in different ways, as they draw the learners’ attention to different aspects of category features (the Sequential Attention Theory; Carvalho and Goldstone 2015a, 2017). Blocking exemplars by category draws learners’ attention to the common features shared among the exemplars. Hence, a blocking schedule has been shown to be more effective than interleaving when the more challenging part of the materials to be learned is to identify the commonalities among exemplars of one concept (Carvalho and Goldstone 2017, 2019; also see Brunmair and Richter 2019). By contrast, an interleaving schedule is more advantageous than blocking in the learning of highly similar, therefore, confusable concepts, because interleaving draws learners’ attention to the critical differences that separate one category from another (i.e., the discriminative-contrast hypothesis; Birnbaum et al. 2013; Kang and Pashler 2012). Indeed, the facilitating effect of interleaving, referred to as the *interleaving effect*, has been supported in many kinds of highly confusable categories, from natural rock categories (Whitehead et al. 2021), bird and butterfly species (Birnbaum et al. 2013; Wahlheim et al. 2011) to scientific concepts in mathematics (Rohrer et al. 2015), chemistry (Eglington and Kang 2017), and statistics (Sana et al. 2017).

Further, interleaving exemplars of different categories also creates a time gap or a temporal spacing between the successive presentation of two exemplars from the same category. This time gap requires learners to exert more cognitive effort in retrieving previously learned information. Such extra effort can, in turn, strengthen the memory trace and improve the retention of that information in the long-term (i.e., the *spacing effect*; see Carpenter 2014; Carpenter et al. 2012 for reviews).

Research demonstrates that the attention- and memory-based accounts of the interleaving effect can interact and together promote category learning. Specifically, under an interleaving schedule, learners are more likely to notice the unique features of a category as learners pay more attention to between-categories differences. Those features, once identified, can be remembered better due to the memory benefits of an interleaving schedule (i.e., the two-stage framework of sequencing effects; Yan et al. 2020; Yan and Schuetze 2022).

Study schedules have also been shown to affect different category learning processes. Noh et al. (2016) found that blocked schedule promoted rule-based category learning better than interleaving because blocking exemplars by category allows learners to test their hypotheses about the underlying pattern of one category at a time. In contrast, an interleaved schedule can improve information-integration category learning because it encourages students to define category membership more comprehensively in relation to other categories.

Interestingly, blocking is believed by many learners to be more effective than interleaving schedule even when the empirical evidence shows the opposite (see Kang 2016 for an overview). For example, Kornell and Bjork (2008) had participants learn painting styles of twelve different artists through a series of their paintings. For half of the artists, their paintings were arranged in a blocked manner across the study, and for the other half, the paintings were interleaved. Participants later took a classification test in which they were presented with new paintings and had to identify the artists who painted the novel paintings. Results showed that the interleaving schedule led to better identification accuracy as compared to blocking. Interestingly, only 22% of the participants accurately judged interleaving to be more effective than blocking, suggesting that the interleaving benefits seem counterintuitive. This phenomenon has been replicated many times in the literature for a wide range of study materials, including both visual (e.g., Birnbaum et al. 2013; Kornell and Bjork 2008; Kornell et al. 2010) and text stimuli (e.g., Zulkiply et al. 2012; Zulkiply and Burt 2013).

However, different types of metacognitive judgments may result in different findings. For example, using the same paintings materials, Yan et al. (2016) found that when learners made category-learning judgments, they reported higher confidence in their ability to classify new exemplars of the interleaved categories than of the blocked categories. However, similar to Kornell and Bjork’s (2008) findings, the majority of participants selected blocking to be the more effective study schedule. The selection of the more effective study schedule can be biased because it is affected by both participants’ prior knowledge and their sense of fluency when learning, thus leading to more erroneous metacognitive judgments (Yan et al. 2016). Further, even when participants were aware of improved learning for the interleaved categories, many participants did not attribute better learning to the benefits of the interleaving schedule but to other irrelevant factors (e.g., the relative difficulty level of the categories or luck), and thus did not appreciate the interleaving effect (Yan et al. 2016).

It is worth noting that Yan et al. (2016) used a within-participant experimental design, which can increase the accuracy of metacognitive predictions as participants can compare cues that result from one condition of the study to the cues associated with another condition. The same results may not be observed in a between-participants design. The present study sought to further investigate the impact of study schedules on category learning and metacognitive judgment when study schedules are combined with feature descriptions.

### 2.2. Research on Feature Descriptions

The question of the value of feature descriptions is important, especially when it comes to the learning of natural concepts such as rock categories. Natural rock categories possess complex structures and are sometimes difficult to differentiate and articulate (Nosofsky et al. 2017). They differ from well-defined categories in scientific domains (e.g., mathematics, statistics, or chemistry) as well as artificial laboratory categories because natural rock categories exhibit greater variations across multiple dimensions. Within each dimension, many rock features demonstrate continuous variation rather than being characterized by discrete features (e.g., blue vs. yellow vs. red colors; Nosofsky et al. 2017). Providing feature descriptions (generated by experts in geoscience education) may facilitate category distinction because they can draw learners’ attention to relevant dimensions that separate exemplars of different rock categories. On the other hand, feature descriptions may not benefit all rock categorization because learning the exemplars along with feature descriptions may bias learners’ attention to some specific dimensions rather than encouraging them to process the rock exemplars in a more complex, multi-dimensional space (Miyatsu et al. 2019).

Much research has examined the effects of providing feature descriptions on rock category learning, but empirical evidence has been inconclusive. Presenting feature descriptions may or may not improve learning, depending on the learning tasks and study materials. Miyatsu et al. (2019) investigated the effect of providing feature descriptions in a series of rock categorization experiments. They had two groups of participants learn exemplars from several rock categories in an interleaved order and compared learning in the presence or absence of verbal descriptions. Results showed that verbal descriptions improved category learning when the features were visually highlighted and linked directly to aspects of specific images. When feature descriptions were provided separately below the image, they did not improve category learning.

The benefit of providing feature descriptions, however, was not replicated in a later study by Meagher et al. (2022) using the same learning task and materials. Meagher et al. (2022) only found the benefits of highlighting feature descriptions when they changed the materials to a series of rock pairs that were highly confusable from one another (e.g., Anthracite vs. Obsidian, Basalt vs. Hornfels, etc.). Importantly, Meagher et al. (2022) also suggest that, among the rock categories, some can be differentiated based on a few discrete features with regard to one or two specific dimensions. Specifically, Meagher et al. (2022) had participants rate each rock exemplars along several dimensions and found that some rock exemplars were easier to differentiate along these explicit dimensions while others were not. These results suggest that within the broad category of rocks, some sub-categories may be categorized using rule-based criteria while others require a more comprehensive integration of information across multiple dimensions. Given such possible differences in category learning processes, it is important to examine the effect of study schedule and feature descriptions in the learning of rule-based versus information-integration category learning.

Verbal descriptions of features that define a category membership are prevalent in geoscience textbooks and laboratory manuals (e.g., Cronin 2018; Marshak 2019; Tarbuck and Lutgens 2018). However, research revealed that providing feature descriptions is not always beneficial in rock category learning (Kang et al. 2023; Meagher et al. 2022; Whitehead et al. 2021). We sought to further understand its impact on category learning by examining the effect of providing feature descriptions on participants’ metacognitive judgment. To the best of our knowledge, there have not been any studies yet to investigate how explicitly providing verbal explanation of category characteristics may affect participants’ metacognitive assessment of their learning. When feature descriptions are not provided, participants may be required to invest more effort to abstract the category features that represent a category membership. Learners may misinterpret higher effort as poor learning (de Bruin et al. 2023; Onan et al. 2022), and thus feel less confident of their learning status. For that reason, when feature descriptions are explicitly provided, regardless of their actual benefits on category distinction, participants may feel more confident about their learning, thus predicting higher test performance. Providing feature descriptions may generate a metacognitive illusion that explicit verbal explanations enhance learning even when they do not. Such a misbelief can lead to nonoptimal study behaviors which may impair learning eventually.

### 2.3. Feature Descriptions Interact with Study Schedule

One major interest of the present study was to examine how explicitly providing feature descriptions may affect the effect of interleaving and blocking study schedules. Some previous studies can contribute to answer this question using different learning materials. For example, in a learning task of science categories (i.e., organic chemical compounds), Eglington and Kang (2017) found the robust benefits of interleaving schedule over blocking both when the rules that define category membership were and were not visually highlighted on the exemplars. Using a learning task of Chinese characters, Yan and Schuetze (2022) also found the positive effect of interleaving schedule, but this only emerged when the category-level rules were visually highlighted to the participants. No benefits of interleaving were found when the rules were not provided. It appears that when the rules are difficult to notice, interleaving alone was not enough to promote better category learning. Rather, these results suggest that category learning may improve when interleaving is paired with feature descriptions, at least in situations where features are easy to verbalize and diagnostic of category membership.

Altogether, previous findings demonstrate the possible interaction between the effect of study schedule and understanding diagnostic features for category learning. However, less research has investigated the relationship between these two factors in naturalistic category learning. One recent study, using Miyatsu et al. (2019)’s rock materials, found that feature descriptions did not offer any benefit to one study schedule over another in rock categorization (Whitehead et al. 2021). The present study sought to re-examine the effect of study schedule and feature descriptions by having participants learn the confusable rock pairs from Meagher et al. (2022). Meagher et al. (2022) found that some rock pairs contain discrete features that are relatively easier to verbalize, while others do not. If feature descriptions are beneficial for rock category learning, providing them may boost the interleaving effect as they draw learners’ attention to the relevant dimensions when learners compare exemplars from different categories (Yan and Schuetze 2022). Further, interleaving schedule can also enhance learning as it improves learners’ memory of the provided features (Yan et al. 2020; also see Carpenter 2014). By using the rock materials from Meagher et al. (2022), we aimed to offer more insights into the possible interaction between study schedule and feature descriptions in different learning processes.

## 3. The Present Study

We examined the effect of study schedule and feature descriptions on rock category learning and metacognitive predictions of learning in different settings across three experiments. We used the rock-pair materials and feature descriptions from Meagher et al. (2022). We divided the six rock pairs into three rule-based rock pairs and three information-integration rock pairs. The rule-based rock pairs contain discrete features that can differentiate or partially differentiate exemplars of the two rock categories. Thus, feature descriptions that draw learners’ attention to these relevant features were diagnostic of category membership. However, for the three information-integration rock pairs, feature descriptions were not diagnostic of category membership (Meagher et al. 2022). For these categories, we expected participants to benefit from interleaving but not necessarily from feature descriptions.

The purpose of Experiment 1 was to examine the effect of study schedule and feature descriptions as well as the interaction between the two factors in rule-based and information-integration category learning. In Experiments 2 and 3, we focused only on information-integration category learning and varied the way exemplars were presented to the participants. We also examined the effect of study schedule and feature descriptions on participants’ predictions of future performance in Experiments 2 and 3. We aimed to illuminate the impact of study schedules and of providing feature descriptions on participants’ metacognitive assessment of their learning.

## 4. Experiment 1

### 4.1. Method

#### 4.1.1. Design

Our study was approved by the Institutional Review Board (IRB) of Tufts University. We adopted a 2 × 2 × 2 mixed design with study schedule (interleaving vs. blocking) and feature descriptions (FD present vs. FD absent) being manipulated between participants, resulting in four different conditions. We had all the participants learn a series of rock images from the twelve different rock categories adopted from Meagher et al. (2022). Categorization processes (rule-based vs. information-integration learning) were manipulated within participants, meaning that participants learned all the twelve rock categories.

#### 4.1.2. Participants

A priori power analysis was conducted using G*Power 3.1 (Faul et al. 2007) to determine the smallest sample size required for a 2 × 2 between-participants analysis of Variance (ANOVA) with the power being set at 0.95, α = 0.05, and Cohen’s f = 0.20. The analysis suggested a sample size of 327 but we ended up recruiting 457 participants via Prolific, an online platform for participant recruitment. We anticipated some level of attrition due to technical problems and other unexpected situations that participants could possibly encounter given the online platform.

All the participants in our sample were residing in the USA and were older than 18 years old at the time they participated in this study. The sample was stratified in terms of age, sex and ethnicity^1^ so that it represented the same proportion of the USA population. This is referred to as a representative sample on Prolific. Data from 65 participants were eliminated from data analysis. Among them, 16 participants did not have a normal or corrected to normal vision. Twenty-one participants inaccurately identified one or two items presented for attention check and one participant encountered a technical problem during the study. Lastly, 27 participants showed some level of interruption during the study as the total time they spent on the study were considered outliers based on the boxplot of the data set (i.e., exceeding 70 min = upper quartile + 1.5 × interquartile range; *Mdn* = 41 min). Elimination was not significantly predicted by condition; χ^2^ = 0.85, *df* = 1, *p* = .444.

This elimination process led to a final sample of 392 participants for data analysis (*M*_age_ = 44.94, *SD*_age_ = 15.95, age range from 18 to 84, 50% Female, 71% White) with 91 participants in the FD absent, interleaving condition; 109 in the FD absent, blocking condition; 110 in the FD present, interleaving condition; and 82 in the FD present, blocking condition.

#### 4.1.3. Materials

We adopted a series of 144 rock images, representing 144 rock exemplars, from 12 different rock categories (i.e., Anthracite, Basalt, Breccia, Conglomerate, Gabbro, Hornfels, Marble, Micrite, Obsidian, Peridotite, Rock gypsum, and Shale) from Meagher et al.’s (2022) study. The 12 rock categories consisted of 6 different rock pairs with each pair containing 2 rock categories that were deemed to be highly confusable from one another. The rock pairs were generated by experts in the field of geoscience. Table 1 shows the six rock pairs and their feature descriptions. Specifically, Anthracite was paired with Obsidian, Basalt with Hornfels, Breccia with Conglomerate, Gabbro with Peridotite, Marble with Rock gypsum, and Micrite with Shale. The feature descriptions included some common features that were shared between two members of a rock pair and some distinctive features to distinguish one member from another. For example, for the Anthracite/Obsidian pair, Anthracite was described as dark, black, and shiny with rough, layered surfaces, whereas Obsidian was also described as dark, black, and shiny but with smooth, scalloped surfaces (refers to Table 1).

The Anthracite/Obsidian, Breccia/Conglomerate, Gabbro/Peridotite pairs were assigned to be the sub-categories that should benefit from rule-based learning. Based on participants’ ratings in Meagher et al. (2022), Anthracite/Obsidian exemplars appeared to have clearly separated distribution in the rough/smooth surfaces dimension. Breccia/Conglomerate also had clear distinction in terms of angular/rounded fragments. For Gabbro/Peridotite, green tinting was a distinctive feature of Peridotite exemplars. One Gabbro exemplar was rated 7 (out of 9) in terms of green tinting, which may be highly confusable with Peridotite (Meagher et al. 2022). However, given the complex structure of natural categories compared to artificial or scientific learning materials, we believed that Gabbro/Peridotite was relatively more rule-based and can be partially differentiated based on the provided rules compared to other rock pairs. Further, having a green tinting is a discrete feature that can be relatively easy to verbalize.

The Basalt/Hornfels, Marble/Rock gypsum, and Micrite/Shale were assigned to be the sub-categories that benefit from information-integration learning. There was much overlapping distribution of feature ratings in the dimensions that the expert geoscientist explicitly pointed out to be the differences of the Basalt/Hornfels and Marble/Rock gypsum pairs (Meagher et al. 2022). There was no report of feature ratings for Micrite/Shale in Meagher et al. (2022). However, the classification accuracy for this pair was relatively lower than other rock pairs (with or without feature descriptions). This indicates that it was difficult to verbalize the distinctive features of Micrite/Shale.

All the feature descriptions were presented as key words, instead of full sentences to avoid inducing extraneous cognitive load on subjects’ working memory^2^ (Mayer 2002, 2005). We randomly assigned 24 exemplars (2 exemplars × 12 rock categories) to the pretest, 72 exemplars (6 exemplars × 12 rock categories) to the study, and 48 exemplars (4 exemplars × 12 rock categories) to the final transfer test. There were no overlapping rock stimuli across the pretest, the study, and the final test.

#### 4.1.4. Procedure

Participants accessed the study via Prolific and then were redirected to Qualtrics to participate in the experiment. First, we had them take a pretest to measure their prior knowledge of the to-be-learned rock categories. This is a classification test asking participants to identify the rock category of several rock exemplars, given one at a time. They selected their answers from a list of 12 rock categories (i.e., the to-be-learned rock categories) presented in alphabetical order. Participants were asked not to use outside sources while taking the test. There was no time limit, and feedback was not provided.

After the pretest, participants were instructed that they will be learning twelve different rock categories through a series of exemplars, and then later take a test on how well they can identify the category of new exemplars. In addition, participants were informed beforehand that there would be some images of unrelated objects randomly presented during the study to check if they were paying attention to the study. They were instructed to quickly write down the unrelated objects when they popped up (e.g., a bowl, a pencil, etc.) because they would be asked what those objects were after they finished the study phase. Participants were also explained that they would not have to remember any other details about the unrelated objects and were asked not to take notes during the study, except for the attention-check trials.

Table 2 shows examples of the first six trials from the FD absent, interleaving and FD absent, blocking conditions (A) and an example of a trial from the FD present conditions (B). We had the participants passively study the individual exemplars, meaning that every exemplar was presented along with its corresponding category name. One exemplar was presented at a time for six seconds and were arranged in an interleaved or a blocked order depending on the conditions. Also, the feature descriptions of the corresponding rock category were provided along with the rock image or not provided at all, according to the conditions. The feature descriptions, if available, were provided below the rock image (see Table 2B).

Under the interleaved schedule, all study stimuli were divided into six blocks with a block containing one exemplar from each of the 12 categories. The rock exemplars were presented in a fixed randomized order in which no exemplar from the same category were presented consecutively (refers to Table 2A). We presented the participants with the whole six blocks and then repeated them again in the same order (i.e., 12 exemplars × 6 blocks × 2 times).

Under the blocked schedule, a study block consists of all the six exemplars of one category. The six exemplars in a block were presented to the participants one by one (refer to Table 2A), and then the whole block was repeated again in the same order. After studying the exemplars of one category twice, participants were moved to study another category (i.e., 6 exemplars × 2 times × 6 blocks).

Two irrelevant images of a chair and a fork were presented randomly during the study phase for attention check. After finishing the study, participants were asked to identify these two unrelated objects among a list of ten different objects (e.g., a table, a chair, a book, a radio, etc.).

We then had the participants complete a simple calculation task, containing 35 addition and subtraction problems, for five minutes (e.g., 35 + 19 = ?). They were asked not to use any calculator. Participants were automatically forwarded to the following screen after five minutes. Participants were instructed that they would now take an identification test on the twelve rock categories, which they just had learned, and that there would be no time limit and no feedback. Participants were asked not to consult outside sources. The format of the final test was maintained to be the same as the pretest. All the final test items were novel rock images that participants did not encounter in the pretest nor in the study phase, making it a transfer test.

After finishing the final transfer test, participants were asked to answer a few survey questions, about if they have a normal or corrected to normal color vision, their age, gender identity, and race/ethnicity. Finally, participants were thanked for their participation in the present study and were rewarded approximately USD 8 per hour for their participation.

#### 4.1.5. Data Analysis

Experiment 1 sought to examine the effect of study schedule and feature descriptions on the learning of rock categorization. We did not predict a three-way interaction between study schedule, feature descriptions, and different learning processes. Interleaved study and feature descriptions have potential benefits to promote rock categorization through both rule-based and information-integration learning (Meagher et al. 2022; Miyatsu et al. 2019; Whitehead et al. 2021). The present study aimed to examine the extent to which interleaving schedule and feature descriptions can facilitate the two learning processes.

We took out two subsets of data according to the two different learning processes that the rock categories may elicit (rule-based vs. information-integration). For each subset, we conducted a logistic generalized linear mixed-effects model (GLMM) analysis to predict whether participants correctly or incorrectly identified the rock category of a given rock image on each trial. We included study schedule (interleaving coded as 1 vs. blocking as 0), feature descriptions (FD present as 1 vs. FD absent as 0), and their two-way interaction as the predictors. Participants’ classification accuracy for each rock category in the pretest was also included in the model as a covariate. Furthermore, we incorporated two random effects for the interception to our model: one for participants (1|Participant ID) and another one for items nested within rock categories (1|Rock Category/Item) to account for the random variation across participants and across test items, respectively. We estimated the model using the *lme4* packages in R (Bates et al. 2015).

We performed Wald chi-square tests, using Type III ANOVA function in R, to evaluate the significance of the effects of the predictors. We also report Hedges’ *g* and the odds ratios (*OR*) to interpret the effect size of the predictors. An *OR* larger than 1 indicates that a one-unit higher score on the predictor is associated with higher odds of correct identification. An *OR* smaller than 1 demonstrates that a one-unit higher score on the predictor is associated with lower odds of correct identification. When an *OR* is not significantly different than 1, the odds of correct identification do not vary as a function of the predictor variable.

### 4.2. Results

**Figure 1:**
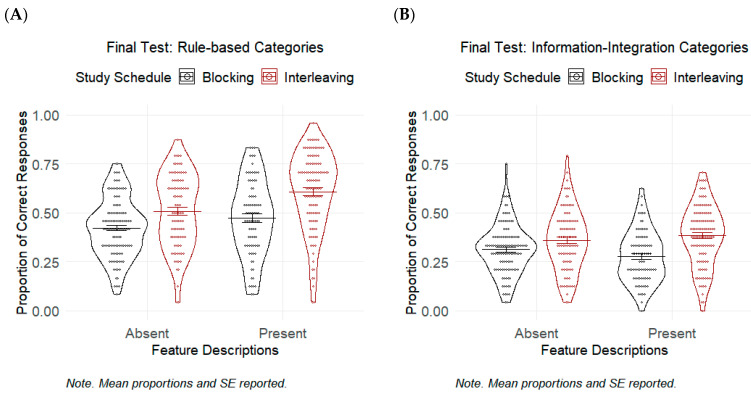
The final classification test performance as a function of study schedule (interleaving vs. blocking) and feature descriptions (FD absent vs. FD present) in Experiment 1 (12 rock categories). In (**A**,**B**) are the final classification test performance for rule-based and information-integration category learning, respectively. The horizontal bars represent the mean accuracy proportions. The data points indicate the distribution of individual performance across the sample. The errors bars indicate standard errors (SE).

Table 3 shows the means and standard deviations of the final classification test performance in Experiment 1. The results are also displayed in Figure 1.

#### 4.2.1. Rule-Based Category Learning

We used dummy coding for the predictor variables. Therefore, the logistic GLMM analysis on the final classification test accuracy illustrated the main effect of one predictor variable when it was equal to 1 while the value of the other predictor was equal to 0 (e.g., the effect of interleaving schedule when feature descriptions were absent). Specifically, the results demonstrated the significant effect of study schedule on rock category identification accuracy when feature descriptions were absent: χ^2^(1) = 10.47, *p* = .001, *g* = 0.53, *OR* = 1.60, and 95% CI [1.20, 2.12]. This means that the participants who studied under the interleaved schedule were 1.60 times more likely to classify novel exemplars into one of the studied rock categories than those who studied under the blocked schedule, when feature descriptions were not provided (*M* = 0.51, *SD* = 0.19, and *M* = 0.42, *SD* = 0.15, respectively; refer to Table 1). The effect of providing feature descriptions was also significant: χ^2^(1) = 4.20, *p* = .040, *g* = 0.30, *OR* = 1.36, and 95% CI [1.01, 1.82]. This indicates that participants were 1.36 times more likely to identify the correct rock category when they were provided with feature descriptions (*M* = 0.47, *SD* = 0.19) relative to when they were not provided with feature descriptions (*M* = 0.42, *SD* = 0.15), under the blocked schedule. The interaction between study schedule and feature descriptions was not significant: χ^2^(1) = 1.97 and *p* = .160.

#### 4.2.2. Information-Integration Category Learning

The logistic GLMM analysis showed that the interaction between study schedule and feature descriptions was significant: χ^2^(1) = 3.92, *p* = .048, *OR* = 1.34, and 95% CI [1.003, 1.78]. This suggests that the effect of study schedule varied depending on if the feature descriptions were provided or not. Several follow-up contrasts were performed to shed light on their interaction.

When we did not provide feature descriptions, the odds that a participant identified the correct rock category was not significantly higher in the interleaving condition (*M* = 0.36, *SD* = 0.16) compared to the blocking condition (*M* = 0.31, *SD* = 0.13): *OR* = 1.29 and 95% CI [0.996, 1.68]. However, when feature descriptions were provided, participants under the interleaving condition (*M* = 0.38, *SD* = 0.16) were more likely to identify the correct rock category than those under the blocking condition (*M* = 0.28, *SD* = 0.13): *g* = 0.68, *OR* = 1.73, and 95% CI [1.32, 2.27].

Providing feature descriptions did not increase the odds of correct classification, when the exemplars were blocked (*M* = 0.28, *SD* = 0.13 for the FD present, blocking condition vs. *M* = 0.31, *SD* = 0.13 for the FD absent, blocking condition): *OR* = 0.83 and 95% CI [0.63, 1.10]; or when the exemplars were interleaved (*M* = 0.38, *SD* = 0.16 for the FD present, interleaving condition vs. *M* = 0.36, *SD* = 0.16 for the FD absent, interleaving condition): *OR* = 1.11 and 95% CI [0.86, 1.44].

#### 4.2.3. Further Analysis of Individual Rock Categories

We generated a confusion matrix that reveals participants’ responses for each rock category. Appendix A shows the confusion matrix table of participants’ responses in the final classification test of Experiment 1. These results are also presented in Appendix B. We created the confusion matrix only for exploration purposes, without conducting any statistical tests for significance.

### 4.3. Discussion

#### 4.3.1. The Effect of Study Schedule and Feature Descriptions for Rule-Based Category Learning

The results from Experiment 1 demonstrated the advantage of interleaving schedule over blocking for the rock categories that can benefit from rule-based category learning. Specifically, participants were more likely to correctly classify novel exemplars to one of the studied rock categories after an interleaved study than after a blocked study. Our results also highlighted the benefits of providing feature descriptions on classification accuracy when the rock categories possess discrete features that separate them from other categories. Explicit instructions that directed learners’ attention to these features were beneficial as the provided features were diagnostic of category membership.

#### 4.3.2. The Effect of Study Schedule and Feature Descriptions for Information-Integration Category Learning

For information-integration categories, the interleaving schedule did not lead to better classification performance than the blocking schedule when participants studied the rock exemplars without feature descriptions. The interleaving benefits only emerged under a learning environment in which participants were provided with feature descriptions. Providing feature descriptions to participants, however, did not improve the final classification test performance when the stated features were not diagnostic of category members. This result held true for both blocked and interleaved studies.

Our findings are consistent with the prior literature demonstrating the positive effect of interleaving over blocking on rock categorization (Whitehead et al. 2021). We extended the literature by showing the interleaving benefits for both rule-based and information-integration category learning. Our results also suggest that providing feature descriptions was most useful when the to-be-learned categories contain discrete features that can be verbalized. The stated features are helpful because they are diagnostic of category membership.

However, one limitation of Experiment 1 was the low classification accuracy of the rock categories that required information-integration learning process (see Table 3). Such low performances could have affected our results as they may not demonstrate the true variation between participants according to study schedule and the presence or absence of feature descriptions. Experiment 2 was designed to address this problem.

## 5. Experiment 2

In the present experiment, we aimed to replicate the findings from Experiment 1 while attempting to improve the overall performance of our participants. To achieve these goals, we carried out three main changes to the design of Experiment 1. First, we had participants learn only the six rock categories that can benefit from information-integration category learning process (Meagher et al. 2022). Second, we increased the number of study items during the study phase from six to eight exemplars per category. Third, for the interleaving schedule, we juxtaposed the rock exemplars more often according to rock pairs, instead of randomly intermixing them as in the previous experiment. For instance, an exemplar of Marble was often followed by another exemplar of Rock gypsum as Marble and Rock gypsum were in the same rock pair.

Research suggests that an interleaved schedule enhances learning as it allows participants to compare and contrast the exemplars of different categories, thus facilitating category discrimination (Birnbaum et al. 2013; Eglington and Kang 2017; Kang and Pashler 2012). For that reason, we aimed to boost the interleaving benefits and to improve participants’ final classification test performance by providing them more opportunities to compare and contrast the exemplars of highly confusable rock pairs during the study phase.

Furthermore, we sought to investigate participants’ metacognitive assessment of their ability to correctly classify novel exemplars to one of the studied rock categories. We asked participants to make a global prediction of future classification test performance which can reflect the overall confidence of their learning status. Both study schedule and feature descriptions can impact participants’ prediction of performance because blocking schedule (rather than interleaving) and providing feature descriptions can increase participants’ sense of fluency during learning, thus potentially leading to higher learning confidence under these conditions (Onan et al. 2022). The effects of study schedule on metacognitive judgment are often examined when the category-level rules are not explicitly provided. We aimed to extend the literature by examining how participants’ metacognitive judgment varied as a function of study schedule combined with the presence or absence of feature descriptions.

### 5.1. Method

#### 5.1.1. Design

Similar to the previous experiment, we manipulated study schedule (interleaving vs. blocking) and feature descriptions (FD absent vs. FD present) between participants. In Experiment 2, we focused primarily on the rock categories that required an information-integration category learning process.

#### 5.1.2. Materials and Procedure

We used the same rock images from six different rock categories that involved information-integration category learning as in Experiment 1. The six rock categories were also divided into three different rock pairs: Basalt/Hornfels, Marble/Rock gypsum, Micrite/Shale. We increased the number of rock images that participants learned for each rock category by re-presenting the 12 rock stimuli that were used in the pretest again during the study phase (2 exemplars × 6 rock categories). That means, participants learned 48 rock images (8 exemplars × 6 rock categories) in total, instead of 36 rock images as in Experiment 1. Similar to Experiment 1, the rock exemplars were presented one by one for six seconds each. The final test contained only new exemplars and also remained in the same format as in Experiment 1.

Another difference from Experiment 1 was the way we presented the rock exemplars across the study phase in Experiment 2. Specifically, under the interleaved schedule, all the study stimuli were divided into eight blocks, with each block containing six exemplars, one exemplar from each of the six categories. The exemplars were presented one by one for six seconds each, similar to Experiment 1 (see Table 2). For the first and second blocks, all the exemplars were presented according to the pre-determined rock pairs. For example, one exemplar of Basalt was followed by an exemplar of Hornfels, one Marble followed by a Rock gypsum, and then one Micrite followed by a Shale. In the third, fourth, and fifth blocks, we only juxtaposed exemplars by rock pair two times (e.g., Hornfels—Micrite—Shale—Basalt—Marble—Rock gypsum). That means, two exemplars of one rock pair were not juxtaposed (e.g., Hornfels and Basalt). In the sixth, seventh, and eighth blocks, we juxtaposed exemplars by rock pair only one time (e.g., Micrite—Shale—Rock gypsum—Basalt—Marble—Hornfels). In total, exemplars from the same rock pairs are juxtaposed exactly five times across the eight study blocks. We presented participants with the whole eight study blocks and then repeated them again in the same order (i.e., 6 exemplars × 8 blocks × 2 times).

Under the blocked schedule, there were six study blocks with a block consisting of all the eight exemplars of one category. The study exemplars were presented to participants one by one, and then repeated again in the same order. After studying the exemplars of one category twice, participants were moved to study another category (i.e., 8 exemplars × 2 times × 6 blocks).

We had participants make a prediction of future classification test performance after they finished the study phase by reporting how likely they feel they would be able to correctly identify the rock category of new exemplars. The specific instruction was as follows: “In the final test, new examples from the same six rock categories will be presented. How likely do you feel you will be able to correctly put a new rock example in one of the categories that you just learned?” Participants were asked to respond on a 1–4 Likert scale (1 = very unlikely, 4 = very likely). Other than adding the metacognitive question after the study phase, we kept the rest of the procedure similar to Experiment 1.

#### 5.1.3. Participants

We recruited a representative sample of 401 participants from Prolific. Seventy-five participants were removed from data analysis. Among them, 22 participants failed the attention check. Thirty participants reported not to have normal or corrected to normal color vision. Lastly, we removed 23 participants who were considered outliers in terms of the duration time based on the boxplot of our data set (i.e., exceeding 49 min; *Mdn* = 28 min). Elimination was not significantly predicted by condition: χ^2^ = 0.15, *df* = 1, and *p* = .817.

After elimination, we had a final sample of 326 participants for data analysis (*M*_age =_ 43.81, *SD*_age_ = 14.79, age range from 18 to 82, 58% Female, 73% White) with *n* = 72 participants in the FD absent, interleaving condition; *n* = 86 in the FD absent, blocking condition; *n* = 88 in the FD present, interleaving condition; and *n* = 80 in the FD present, blocking condition.

### 5.2. Results

**Figure 2:**
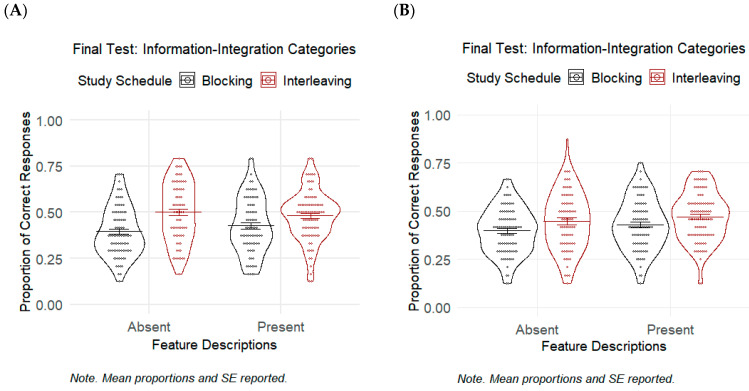
The final classification test performance as a function of study schedule (interleaving vs. blocking) and feature descriptions (FD absent vs. FD present) in Experiment 2 (sequential presentation) (**A**) and Experiment 3 (simultaneous presentation) (**B**). The horizontal bars represent the mean accuracy proportions. The data points indicate the distribution of individual performance across the sample. The errors bars indicate standard errors (SE).

Means and standard deviations of the final classification test performance and metacognitive judgment from Experiment 2 are presented in Table 4. The results are visualized in Figure 2A. We also generated a confusion matrix that reveals participants’ responses for each rock category. Appendix C shows the confusion matrix table of participants’ responses in the final classification test of Experiments 2 and 3.

#### 5.2.1. Final Classification Test Performance

A logistic GLMM analysis was performed to evaluate the effects of study schedule and feature description as well as their interaction on rock category identification accuracy. Results revealed a significant effect of study schedule: χ^2^(1) = 21.10, *p* < .001, *g* = 0.72, *OR* = 1.64, and 95% CI [1.33, 2.03]. This indicates that the interleaved schedule (*M* = 0.50, *SD* = 0.16) increased the odds of accurate rock-category identification by 1.64 times compared to the blocked schedule (*M* = 0.40, *SD* = 0.13) when participants were not provided with feature descriptions. The effect of providing feature descriptions was not significant: χ^2^(1) = 1.92 and *p* = .166. This indicated that the participants who studied feature descriptions along with the rock exemplars under the blocked schedule did not significantly perform better in the final test as compared to those who did were not provided with feature descriptions (*M* = 0.43, *SD* = 0.15 and *M* = 0.40, *SD* = 0.13, respectively). The interaction between study schedule and feature descriptions was not significant: χ^2^(1) = 2.61 and *p* = .106.

#### 5.2.2. Metacognitive Judgment

A 2 × 2 between-subjects ANOVA on participants’ prediction of performance revealed that the main effect of study schedule was not significant: *F*(1, 322) = 0.05 and *p* = .826. This indicated that the participants who studied under the interleaved schedule (*M* = 2.70, *SD* = 0.64) did not feel more confident about their learning as compared to those under the blocked condition (*M* = 2.67, *SD* = 0.69), regardless of the absence or presence of feature descriptions. The main effect of feature descriptions, however, was significant: *F*(1, 322) = 5.73, *p* = .017, and *g* = 0.27. This means that the participants who studied the rock images along with feature descriptions during the study phase felt that they would be more likely to identify the correct rock category of new exemplars in the final test than those who studied without feature descriptions (*M* = 2.77, *SD* = 0.62 and *M* = 2.59, *SD* = 0.71, respectively), regardless of study schedule. The interaction between study schedule and feature descriptions was not significant: *F*(1, 322) = 1.50 and *p* = .221.

### 5.3. Discussion

#### 5.3.1. Final Classification Test Performance

We replicated the findings from the previous experiment and demonstrated that interleaving rock exemplars promoted better rock categorization for information-integration category learning. However, different from Experiment 1, the interleaving effect emerged even when participants were not provided with feature descriptions. The results from Experiment 2 were similar to those of Whitehead et al. (2021), showing that the benefits of interleaving did not vary according to the presence or absence of feature descriptions even when we used highly confusable rock pairs. Furthermore, similar to our results from Experiment 1 and to prior studies, providing feature descriptions did not enhance rock categorization when the rock categories do not seem to have discrete features that can be easily verbalized (Meagher et al. 2022).

#### 5.3.2. Metacognitive Judgment

Our results suggest that the participants who studied under the interleaved schedule were not aware of the interleaving benefits. The interleaved group of participants showed a comparable level of confidence that they would be able to identify the correct rock category of novel exemplars compared to the blocked group. Interestingly, the participants who studied the rock categories along with feature descriptions felt more confident about their ability to classify rock exemplars than those who were not provided with such descriptions. It appears that providing feature description did not enhance the final classification test performance but may lead to inaccurate metacognitive assessment of learning.

The lack of interaction between study schedule and feature descriptions, however, could be due to the way the exemplars were presented during the study phase. In Experiments 1 and 2, only one exemplar was presented on every trial during the study phase. The sequential presentation of rock exemplars did not allow participants to directly compare and contrast the exemplars. The rock categories that require information-integration categorization process is complex and vary across multiple dimensions. Learning a series of isolated exemplars may prevent some participants from interpreting the feature descriptions that we provided. The interaction between study schedule and feature descriptions may become more robust when we make it easier for participants to process the provided information. We designed Experiment 3 to test this hypothesis.

## 6. Experiment 3

We aimed to re-examine the effect of study schedule and feature descriptions in the learning of rock categorization that involved information-integration learning process. Similar to Experiment 2, we had participants learn the same six rock categories. The only difference was that we simultaneously presented two rock images on every trial instead of sequentially presenting them one by one as in previous experiments.

Simultaneous presentation of rock exemplars has been demonstrated to facilitate rock categorization learning (Meagher et al. 2017). Allowing participants to study two stimuli from the same category can emphasize the characteristic features that are shared among the category members (Goldwater and Schalk 2016; Kurtz et al. 2001). On the other hand, allowing participants to simultaneously study the information from two different rock categories may highlight the discriminative features that separate one category from another (Andrews et al. 2011; Carvalho and Goldstone 2014). Given the promising benefits of the simultaneous presentation method, we adopted this format for Experiment 3 and sought to re-investigate the effect of study schedule and feature descriptions on rock categorization.

### 6.1. Method

#### 6.1.1. Materials and Procedure

We used the same series of rock images from Experiment 2. Table 5 demonstrates examples of simultaneous presentation of rock exemplars from Experiment 3. Under the interleaved schedule, the rock images were divided into eight study blocks. Each block contained three trials, with each trial consisting of two exemplars. Every trial was presented for ten seconds. For the first and second blocks, each trial consisted of two exemplars from the two rock categories that were considered a pair (e.g., Marble and Rock gypsum). For the third, fourth, and fifth blocks, two trials followed the rule of those in the first two blocks, while the remaining trial included two rock exemplars from two rock categories that were not in the same pair (e.g., Shale and Basalt). For the sixth, seventh, and eighth blocks, only one trial contained two different exemplars from a rock pair, whereas for the other two trials, two rock images from two unpaired rock categories were presented together. After participants learned all the eight blocks, they studied them again in the same order (i.e., 2 exemplars × 3 trials × 8 blocks × 2 times).

Under the blocked schedule, there were six study blocks. Each block contained all the rock exemplars of a rock category divided into four trials. Each trial consisted of two rock exemplars from the same rock category (e.g., two exemplars from Basalt). After participants learned a block of rock images from one rock category, they studied them again in the same order (i.e., 2 exemplars × 4 trials × 2 times × 6 blocks). The rest of the procedure remained the same as in Experiment 2.

#### 6.1.2. Participants

We recruited a representative sample of 398 participants from Prolific. Fifty-eight participants were removed from data analysis. Among them, 18 failed the attention check. Seventeen participants did not have normal or corrected to normal color vision, and twenty-three participants were considered as outliers in terms of the duration time based on the boxplot of our data set (i.e., spending more than 42 min on the study; *Mdn* = 25 min). Elimination was not significantly predicted by condition: χ^2^ = 0.003, *df* = 1, and *p* = 1.000.

After elimination, we ended up having 340 participants for data analysis (*M*_age =_ 43.25, *SD*_age_ = 15.32, age range from 18 to 77, 56% Female, 74% White). They included *n* = 76 participants in the FD absent, interleaving condition; *n* = 86 in the FD absent, blocking condition; *n* = 94 in the FD present, interleaving condition; and *n* = 84 in the FD present, blocking condition.

### 6.2. Results

Means and standard deviations of the final test performance and metacognitive judgment from Experiment 3 are presented in Table 6. The results are also displayed in Figure 2B.

#### 6.2.1. Final Classification Test Performance

A logistic GLMM model on the classification accuracy revealed a significant effect of study schedule: χ^2^(1) = 5.68, *p* = .017, *g* = 0.37, *OR* = 1.26, and 95% CI [1.04, 1.52]. This implied a benefit of the interleaved schedule over blocking for the participants who did not study with feature descriptions (*M* = 0.45, *SD* = 0.15 and *M* = 0.40, *SD* = 0.12, respectively). The effect of feature descriptions was not significant: χ^2^(1) = 2.60 and *p* = .107. This means that providing feature descriptions did not lead to better classification performance than not providing them under the blocked schedule (*M* = 0.43, *SD* = 0.13 and *M* = 0.40, *SD* = 0.12, respectively). The interaction between study schedule and feature descriptions was not significant: χ^2^(1) = 0.10 and *p* = .753.

#### 6.2.2. Metacognitive Judgment

A 2 × 2 between-subjects ANOVA on participants’ prediction of performance demonstrated that the main effect of study schedule was not significant: *F*(1, 336) = 1.95 and *p* = .164. Results showed that participants reported an equivalent rate of confidence in how likely they would be able to identify the correct rock category of novel exemplars after a blocked (*M* = 2.66, *SD* = 0.67) and an interleaved study (*M* = 2.57, *SD* = 0.66), regardless of the absence or presence of feature descriptions during the study phase. Furthermore, a significant effect of providing feature descriptions was observed: *F*(1, 336) = 5.62, *p* = .018, and *g* = 0.26. This suggested that the participants who studied the rock images along with their feature descriptions felt that they will be more likely to identify the correct rock category of new exemplars in the final test (*M* = 2.70, *SD* = 0.61) than those who were not provided with feature descriptions (*M* = 2.53, *SD* = 0.72), regardless of study schedule. The interaction between study schedule and feature descriptions was not significant: *F*(1, 336) = 0.88 and *p* = .350.

### 6.3. Discussion

#### 6.3.1. Final Classification Test Performance

Our results again highlighted the positive effect of interleaving schedule on rock categorization learning when participants studied two rock images simultaneously on every trial. The interleaved group of participants performed better than the blocked group in the final classification test. Providing feature descriptions did not improve categorization learning nor boost the interleaving effect despite the simultaneous presentation of rock exemplars during the study phase.

#### 6.3.2. Metacognitive Judgment

Even though their final classification accuracy was higher, the participants who studied under the interleaved schedule did not report a higher level of confidence that they would likely identify the correct rock category of novel exemplars as compared to those who studied under the blocked schedule. Furthermore, consistent with the results from Experiment 2, participants felt more confident about their ability to classify novel exemplars when they studied the rock exemplars along with feature descriptions. This finding indicates that participants were at risk of making faulty metacognitive judgments when studying with feature descriptions.

## 7. General Discussion

A great deal of research has examined the effect of study schedule on category induction. We extended the literature by investigating how the effect of interleaving and blocking schedules varied when combined with verbal descriptions of category features. Across three experiments, we manipulated study schedule (interleaving vs. blocking) and feature descriptions (FD present vs. FD absent) as participants studied exemplars of rocks from different categories. In general, we found that interleaving promoted both rule-based and information-integration category learning to a greater extent than blocking, which is consistent with prior studies (Whitehead et al. 2021; also see Brunmair and Richter 2019). Further, we replicated the prior literature showing that feature descriptions facilitated category learning when the categories contain discrete features that are easy to verbalize (Meagher et al. 2022). However, limited benefits of feature descriptions were found when the categories do not possess such distinct features, and thus should benefit from information-integration learning. Finally, our results highlighted participants’ metacognitive biases with regard to the effect of study schedule and feature descriptions.

### 7.1. The Effect of Study Schedule and Feature Descriptions on Rock Categorization

Across the three experiments, we demonstrated the facilitative effect of interleaving (relative to blocking) for both rule-based and information-integration category learning. Interleaved study consistently led to better final classification test performance than blocked study for categories that are best learned through rule-based (Experiment 1) and information-integration learning processes (Experiments 1–3). Our findings are consistent with Whitehead et al. (2021) who found the advantages of interleaved study in rock categorization and with a recent meta-analysis showing robust benefits of interleaved practice for visual stimuli (Brunmair and Richter 2019). In addition, we extended the literature by emphasizing the interleaving effect for a representative sample of participants. The majority of prior research on the interleaving effect investigated college student samples (Brunmair and Richter 2019). Less evidence demonstrating the advantages of interleaving in other age groups such as children or adolescents (e.g., Rau et al. 2013; Rohrer et al. 2014, 2015) and older adults (Kornell et al. 2010) have been found. The present study replicates the positive effect of interleaving across a wide age range throughout the three experiments (i.e., 18–84 years old). We highlight the generalizability of the interleaving benefits for rock categorization learning to a large population of adult learners.

We also replicated the findings that feature descriptions can facilitate rock categorization when the rock categories contain discrete features that can be easily verbalized (Meagher et al. 2022). Providing verbal descriptions of these features can draw learners’ attention to the relevant dimension, which improved the classification accuracy of novel exemplars (Experiment 1). The replication of such benefits is important because the effect of feature descriptions for rock category learning were not consistent and subject to various factors in prior studies (Meagher et al. 2022; Miyatsu et al. 2019). The present study suggested that descriptions of relevant features can foster learning even when they were not visually highlighted on the rock images as in the previous literature (e.g., Meagher et al. 2022; Miyatsu et al. 2019). When the rock categories possess discrete features, the descriptions of these features are diagnostic of category membership. Thus, presenting them verbally alongside rock exemplars can also promote better rock categorization. However, it is worth noting that we only found the benefits of providing diagnostic feature descriptions in Experiment 1 for the rock categories that can be learned best via rule-based category learning. The effect of providing feature descriptions for rock classification was inconclusive when the stated features were not diagnostic of category membership.

For categories that require information-integration learning, participants may benefit from engaging in a more holistic procedural-based process that requires an implicit mapping between rock exemplars and underlying category-level pattern (Ashby and Maddox 2011; Maddox and Ashby 2004; Meagher et al. 2022; Noh et al. 2016). Participants may take advantage of the feature descriptions while integrating information across all the exemplars to develop a comprehensive category representation. However, learning complex information-integration categories may require more training and practice for participants to make sense of the feature descriptions and explicitly recognize the category-level pattern. Providing feature descriptions may still benefit information-integration learning when the stated features are more deeply processed and combined with other effective techniques such as lab-based chemical analyses and microscopic examinations (Meagher et al. 2022).

Alternatively, earlier research suggests that explicitly providing feature descriptions may not facilitate an information-integration learning system (e.g., Ashby and Maddox 2011; Noh et al. 2016). This is because the presence of such information may prevent participants from implicitly associating exemplars with a more complex, unverbalizable pattern—a reflexive, procedural-based process that was found to promote information-integration learning (Ashby and Maddox 2011). Research suggests that when explicit instructions are provided, participants are less likely to think beyond the provided rules and tend to continue using rule-based reflective strategies even when they can learn better from an information-integration reflexive learning process (Chandrasekaran et al. 2016). Furthermore, our participants learned the rock categories through a passive learning paradigm. That means, participants always learned the rock exemplars along with their category label. Passive learning may prompt participants to a partially rule-based learning method (Ashby et al. 1998; Hughes and Thomas 2021). Under a passive learning environment, participants may not be able to abandon a rule-based strategy especially when feature descriptions are explicitly presented on every learning trial. That could be one of the reasons why providing feature descriptions did not facilitate information-integration learning. Our study, however, was not designed to directly test this hypothesis. Further research is needed to examine it in more detail.

The present study also contributed to the literature by investigating how different study schedules may interact with feature descriptions in the learning of rock categorization. There has been insufficient evidence showing the relationship between these two factors in a naturalistic category learning. To the best of our knowledge, Whitehead et al. (2021) was the first to investigate the interaction between study schedule and feature descriptions using the rock materials from Miyatsu et al. (2019). They did not find a significant impact of providing feature descriptions on rock classification and thus eliminated this factor from their analyses. We extended their findings by re-examining the dynamic interaction between study schedule and feature descriptions on the learning of highly confusable rock pairs that may benefit from different learning processes, adopted from Meagher et al. (2022).

### 7.2. The Effect of Study Schedule and Feature Descriptions on Metacognitive Judgment

Our examination of participants’ metacognitive judgments demonstrated that participants were not sensitive to the advantage of interleaved study over blocking on rock categorization. Participants under blocked and interleaved conditions predicted an equivalent likelihood to correctly classify rock exemplars before taking the final test. However, the final test performance showed that the interleaved-group participants achieved higher classification accuracy than did the blocked-group participants.

Much prior research demonstrated participants’ erroneous metacognitive belief that blocking schedule is more effective than interleaving, although actual learning performance demonstrated the opposite (Kornell and Bjork 2008; Kornell et al. 2010; Yan et al. 2016; Zulkiply et al. 2012). Participants may believe that blocking schedule facilitates inductive learning better than interleaving due to their prior knowledge, and also because it may feel easier to study one category at a time than to simultaneously process the examples of multiple categories as in an interleaved schedule (Yan et al. 2016). However, Yan et al. (2016) suggested that participants may be more sensitive to the interleaving benefits when making category-level judgments. We did not replicate such sensitivity when having participants make a global judgment, but we also did not observe a preference towards either direction, blocking or interleaving, in the present study. Importantly, the pair-based confusion matrices for each experiment revealed that the interleaving benefits did not always emerge for all the rock categories. These results suggest that category-level effects may impact learning and likely metacognitive predictions. Future research may elaborate on our findings by having participants make a prediction of how likely they feel they would be able to correctly identify new exemplars of a specific rock category rather than making a global judgment. Further investigation that adopts category-level predictions and that uses statistical techniques which can account for category-level variation may be useful moving forward.

We used highly confusable rock pairs that likely benefited from the interleaving schedule as it promoted comparison of exemplars from different categories. However, participants may not be able to recognize the facilitative effect of interleaving due to its small improvement in terms of classification accuracy compared to the blocking schedule (Rivers et al. 2022). Furthermore, our study had a between-participants design, and thus may not be as effective as a within-participant manipulation to capture the subtle differences associated with the effect of interleaved and blocked study on metacognitive judgment of learning (Carroll 2008). A within-participant design may allow participants to compare from their own experience with both study schedules, and thus may produce more accurate assessment of learning (Carroll 2008; Yan et al. 2016).

The metacognitive predictions also reveal that providing feature descriptions led to higher learning confidence. Adding the descriptions of nondiagnostic features did not enhance classification accuracy but seemed to boost participants’ confidence in their ability to later categorize new exemplars. To the best of our knowledge, our study is the first to show empirical evidence on the effect of feature descriptions on metacognitive judgment. Our study demonstrated a metacognitive risk, in which providing feature descriptions may not always improve classification performance for information-integration category learning but can cause an illusion of learning. Inaccurate metacognitive judgment may have a negative impact on subsequent learning behaviors (Efklides 2014; Metcalfe 2009), thus may eventually hinder learning. The present study highlights the importance of considering metacognitive judgment alongside categorization accuracy when examining the effect of study schedule and feature descriptions in category learning.

### 7.3. Limitations and Suggestions for Future Research

We acknowledge a limitation of our study as we used a passive learning paradigm in which participants learned the rock exemplars along with the name of their rock type. Passive learning may reduce participants’ attention for blocked study because participants learned six exemplars of one rock type consecutively and restudied them again in the same order (e.g., Wahlheim et al. 2011). Thus, the subtle differences in classification performance between the interleaved and blocked groups may be due to a gap in the amount of attention that participants had on their study in the two conditions. However, we reduced the contribution of variable attention by requiring participants to spend a fixed amount of study time on each trial regardless of the conditions. We also removed all the participants who failed to identify the two unrelated objects appearing during the attention-check trials. All participants included in our data analysis correctly pointed out the two unrelated objects even though they were presented randomly across the study phase, thus guaranteeing a comparable level of attention for all those who were included.

Future studies should re-examine the effect of study schedule in rock categorization using a more active learning environment to increase attention for blocked conditions. An active learning environment can also benefit the learning of complex and multi-dimensional categories as it may encourage a procedural thinking process that is effective for an information-integration learning system (Carvalho and Goldstone 2015a; Hughes and Thomas 2021). For example, researchers should allow participants to engage in the study more actively by guessing the rock type of exemplars on every trial (Carvalho and Goldstone 2015a; Meagher et al. 2022). Doing so may motivate participants to think beyond the provided features and search for other discriminate features, thus enhance category distinction (Carvalho and Goldstone 2015a).

### 7.4. Practical Implications for Education

Our study demonstrates the advantage of interleaved study over blocking in rock categorization. We also found the benefits of providing feature descriptions when the given features were diagnostic of category membership and learners could benefit from a rule-based category learning process. We therefore suggest instructors and students to interleave their study for effective learning. Instructors may also provide learners with some descriptions of the diagnostic features that help learners discriminate between the confusable categories. Although not investigated in the present research, previous studies suggest that instructors should create only short verbal descriptions to avoid overloading learners’ working memory (Firth et al. 2021).

However, our study shows that feature descriptions may have limited benefits for learners when the provided features are not diagnostic of category membership. That said, providing feature descriptions may facilitate interleaved learning when participants really struggle and fail to identify the discriminative and characteristic features of complex categories (e.g., Yan and Schuetze 2022). Thus, it is important for instructors and students to keep track of the learning process to execute an appropriate study technique. Further, instructors and students should be aware of the metacognitive consequences of providing feature descriptions. Although studying such information may not always enhance later performance, it may inflate students’ confidence on their learning status. Instructors and students should be aware of this phenomenon to adjust their subsequent teaching and learning behaviors.

## 8. Conclusions

The present study provided evidence showing how study schedule and feature descriptions may affect category induction differently according to different learning environments and learning processes. We extended the literature by showing their impact on both participants’ categorization performance and their metacognitive judgment of their ability to do so. Our results highlighted both the benefits and the risks of using these techniques in category learning. Future study should extend our findings by investigating the effects of study schedule and feature descriptions in other circumstances to better understand their possible interaction and the underlying mechanism of the two factors.

## Figures and Tables

**Table 1:** The six rock pairs and their feature descriptions (FD) including the commonalities and differences between two rock categories of each pair.

	**1a. Anthracite**	**1b. Obsidian**	**2a. Breccia**	**2b. Conglomerate**	**3a. Gabbro**	**3b. Peridotite**
	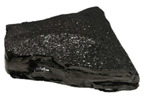	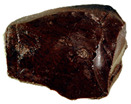	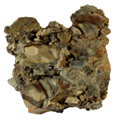	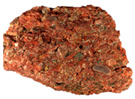	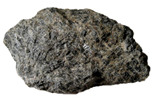	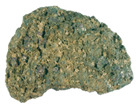
Commonalities	Dark, black, and shiny		Cemented fragments		Dark with coarse-grained crystals	
Differences	Rough, layered surfaces	Smooth, scalloped surfaces	Angular fragments	Rounded fragments		Green tinge
	**4a. Basalt**	**4b. Hornfels**	**5a. Marble**	**5b. Rock Gypsum**	**6a. Micrite**	**6b. Shale**
	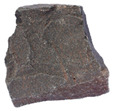	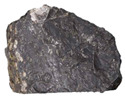	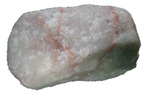	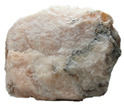	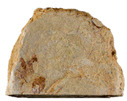	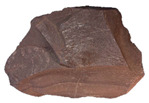
Commonalities	Dark, fine-grained		Light-colored, crystals		Fine-grained	
Differences	May have holes	Layering and flat surfaces	May have interlocking crystals, may have swirling veins	Is often a single large crystal, may be cloudy/translucent	Dense	Often has thin, parallel layers

Note. Adapted from Meagher et al.’s (2022) Table 2 (p. 9) for the FD of the first five pairs and Appendix D for the FD of the last pair (p. 30). The first, second, and third pairs were assigned to be the sub-categories that can benefit from rule-based learning. The fourth, fifth, and sixth pairs were assigned to be the sub-categories that can benefit more from information-integration learning.

**Table 2:** Examples of sequential presentation of rock exemplars from Experiments 1 and 2.

A. FD Absent							B. FD Present
**Interleaving**	Breccia	Gabbro	Obsidian	Micrite	Shale	Rock Gypsum	Breccia
	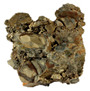	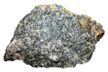	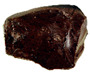	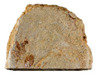	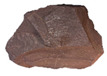	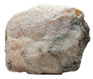	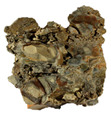
**Blocking**	Basalt	Basalt	Basalt	Basalt	Basalt	Basalt	Cemented fragments, angular fragments
	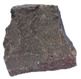	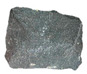	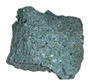	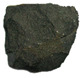	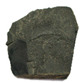	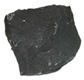	

Note. In (**A**) are the first six trials from the FD absent, interleaving and FD absent, blocking conditions in Experiment 1. In (**B**) is an example of a trial from the FD present conditions in Experiment 1. FD indicates feature descriptions.

**Table 3:** The final classification test performance according to four different conditions in Experiment 1.

Feature Descriptions	Study Schedule	Rule-Based Category Learning	Information-Integration Category Learning
FD Absent	Interleaving	0.51 (0.19)	0.36 (0.16)
	Blocking	0.42 (0.15)	0.31 (0.13)
FD Present	Interleaving	0.61 (0.20)	0.38 (0.16)
	Blocking	0.47 (0.19)	0.28 (0.13)

Note: In the parentheses are the standard deviations of the means. FD indicates feature descriptions.

**Table 4:** The final classification test performance and metacognitive judgment according to four different conditions in Experiment 2.

Feature Descriptions	Study Schedule	Final Test	Metacognition
FD Absent	Interleaving	0.50 (0.16)	2.65 (0.70)
	Blocking	0.40 (0.13)	2.55 (0.71)
FD Present	Interleaving	0.48 (0.14)	2.74 (0.60)
	Blocking	0.43 (0.15)	2.81 (0.64)

Note: In the parentheses are the standard deviations of the means. FD indicates feature descriptions.

**Table 5:** Examples of simultaneous presentation of rock exemplars from Experiment 3.

A. FD Absent							B. FD Present	
**Interleaving**	Trial 1		Trial 2		Trial 3		Trial 1	
	Basalt	Hornfels	Marble	Rock Gypsum	Micrite	Shale	Basalt	Hornfels
	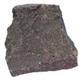	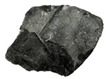	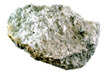	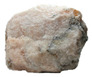	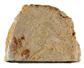	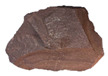	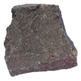	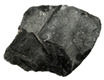
**Blocking**	Trial 1		Trial 2		Trial 3		Dark, fine-grained, may have holes	Dark, fine-grained with layering and flat surfaces
	Basalt	Basalt	Basalt	Basalt	Basalt	Basalt		
	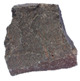	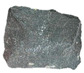	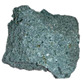	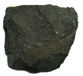	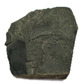	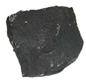		

Note. In (**A**) are the examples of the first three trials from the FD absent, interleaving and FD absent, blocking conditions. In (**B**) is an example of the first trial from the FD present, interleaving condition. FD indicates feature descriptions.

**Table 6:** The final classification test performance and metacognitive judgment according to four different conditions in Experiment 3.

Feature Descriptions	Study Schedule	Final Test	Metacognition
FD Absent	Interleaving	0.45 (0.15)	2.51 (0.72)
	Blocking	0.40 (0.12)	2.55 (0.73)
FD Present	Interleaving	0.47 (0.13)	2.62 (0.61)
	Blocking	0.43 (0.13)	2.79 (0.60)

Note: In the parentheses are the standard deviations of the means. FD indicates feature descriptions.

**Figure A1:**
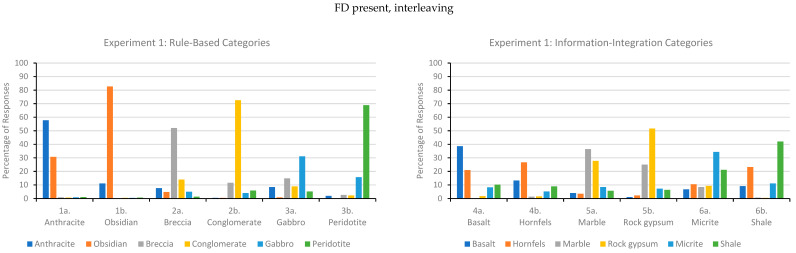

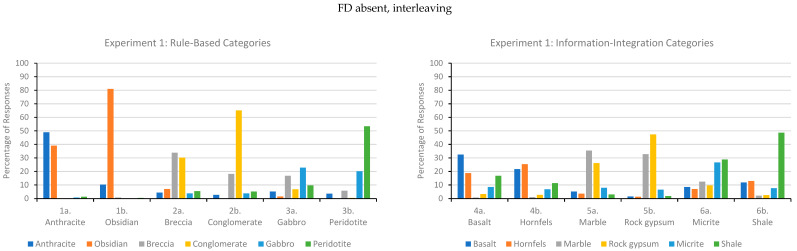

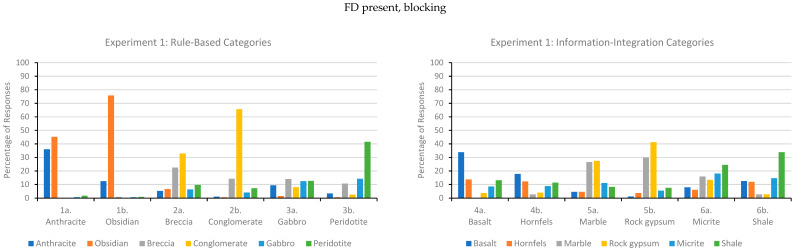

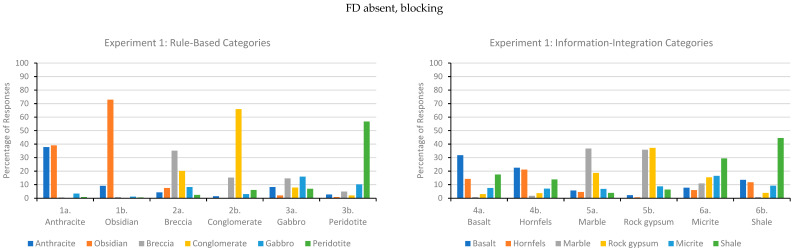
The confusion matrix showcasing participants’ responses in the final classification test from Experiment 1.

## Data Availability

Data are available in Open Science Framework at https://osf.io/hdyt7/.

## Notes

1 The sample was divided into different subgroups according to age (i.e., the age range 18–27, 28–37, 38–47, 48–57, and 58–150), sex (i.e., female and male) and ethnicity (i.e., White, Black, Asian, Mixed, and Other).

2 The feature descriptions for the Micrite–Shale pair were originally explained in full sentences in Meagher et al. (2022). In the present study, they were edited and presented to participants as key words (i.e., Micrite as “*fine-grained, dense*” and Shale as “*fine-grained, often has thin, parallel layers*”).

## Appendix A

**Table A1:** The confusion matrix showcasing participants’ responses in the final classification test of Experiment 1.

FD present, interleaving													
		Experiment 1: Rule-Based Categories						Experiment 1: Information-Integration Categories					
		**1a. Anthracite**	**1b. Obsidian**	**2a.** **Breccia**	**2b. Conglomerate**	**3a. ** **Gabbro**	**3b. Peridotite**	**4a. ** **Basalt**	**4b. ** **Hornfels**	**5a. ** **Marble**	**5b. Rock gypsum**	**6a. ** **Micrite**	**6b. ** **Shale**
Participants’ Responses	Anthracite	**57.7**	**11.1**	7.7	0.5	8.4	2	5.2	7	1.1	0.2	1.4	3.6
	Obsidian	**30.7**	**82.7**	4.8	0.5	0.9	0	0.7	2	1.4	3	0.9	0.2
	Breccia	0.9	0.2	**52**	**11.6**	14.8	2.7	3.6	3.2	1.6	0.7	1.1	1.8
	Conglomerate	0.7	0.5	**13.9**	**72.5**	8.9	2.3	0.2	0.5	1.4	0.2	1.1	1.6
	Gabbro	0.9	0.5	5	4.1	**31.1**	**15.7**	9.3	15.5	8	1.8	3.4	4.8
	Peridotite	1.1	0.7	1.4	5.9	**5.2**	**68.9**	1.1	15	0.7	0.5	1.6	1.4
	Basalt	1.8	0.5	2	1.6	15	2	**38.6**	**13.2**	4.1	1.1	6.8	9.1
	Hornfels	2.7	0.2	2	0.9	7.5	1.8	**20.9**	**26.6**	3.6	2.3	10.5	23.2
	Marble	0	1.6	4.5	0.7	0.5	1.1	0	1.4	**36.4**	**25**	8.4	0.7
	Rock gypsum	0.9	0.2	3	0.9	2	0.9	1.8	1.6	**27.7**	**51.6**	9.3	0.5
	Micrite	1.4	1.1	2.3	0.5	3.9	2.5	8.2	5.2	8.4	7.3	**34.3**	**11.1**
	Shale	1.1	0.7	1.4	0.5	1.8	0	10.2	8.9	5.7	6.4	**21.1**	**42**
FD absent, interleaving													
		Experiment 1: Rule-Based Categories						Experiment 1: Information-Integration Categories					
		**1a. Anthracite**	**1b. Obsidian**	**2a.** **Breccia**	**2b. Conglomerate**	**3a.** **Gabbro**	**3b. Peridotite**	**4a. ** **Basalt**	**4b.** **Hornfels**	**5a.** **Marble**	**5b. Rock gypsum**	**6a.** **Micrite**	**6b. ** **Shale**
Participants’ Responses	Anthracite	**48.9**	**10.2**	4.4	2.7	5.2	3.6	4.7	5.8	3	2.2	0.3	3.8
	Obsidian	**39**	**81**	7.1	0.3	1.6	0.3	1.6	1.1	0.3	1.6	0.5	0.8
	Breccia	0	0.8	**33.8**	**18.1**	16.8	5.8	1.4	3.6	2.5	1.4	1.9	3
	Conglomerate	0.3	0	**30.2**	**65.1**	6.9	0.5	1.4	0.8	0.8	0.3	0.5	1.1
	Gabbro	0.8	0	3.8	3.8	**22.8**	**20.1**	8	12.9	8	1.4	2.7	4.4
	Peridotite	1.4	0.5	5.5	5.2	**9.6**	**53.3**	2.5	6.9	4.1	1.6	0.8	1.1
	Basalt	1.1	1.9	1.6	0.8	16.5	5.8	**32.4**	**21.7**	5.2	1.6	8.5	11.8
	Hornfels	3	0.5	2.2	0.3	12.4	3	**18.7**	**25.3**	3.6	1.4	7.1	12.9
	Marble	0.8	1.4	5.8	0.5	0.5	1.9	0.8	1.1	**35.4**	**32.7**	12.4	2.2
	Rock gypsum	0.3	1.1	2.2	1.9	2.2	2.5	3.3	2.7	**26.1**	**47.3**	9.6	2.5
	Micrite	2.5	1.4	1.9	1.1	4.4	3	8.5	6.9	8	6.6	**26.6**	**7.7**
	Shale	1.9	1.1	1.4	0	1.1	0.3	16.8	11.3	3	1.9	**28.8**	**48.6**
FD present, blocking													
		Experiment 1: Rule-Based Categories						Experiment 1: Information-Integration Categories					
		**1a. Anthracite**	**1b. Obsidian**	**2a.** **Breccia**	**2b. Conglomerate**	**3a.** **Gabbro**	**3b. Peridotite**	**4a. ** **Basalt**	**4b.** **Hornfels**	**5a.** **Marble**	**5b. Rock gypsum**	**6a.** **Micrite**	**6b. ** **Shale**
Participants’ Responses	Anthracite	**37.8**	**9.1**	4.3	1.5	8.2	2.7	7.3	9.8	3.4	4	4	5.2
	Obsidian	**39**	**72.9**	7.6	0	2.1	0.9	1.8	1.5	1.2	0.9	0.9	2.7
	Breccia	0.6	0.9	**35.1**	**15.2**	14.6	4.9	4.9	7	2.4	0.9	2.7	4.3
	Conglomerate	0.3	0	**20.1**	**65.9**	7.9	2.1	0.9	0.3	0.3	0.9	0.3	1.5
	Gabbro	3.4	1.2	8.2	3	**15.9**	**10.1**	9.1	12.5	7.9	2.7	3.7	4.9
	Peridotite	0.9	0.6	2.4	6.1	**7**	**56.7**	2.7	12.2	2.4	1.5	2.7	3
	Basalt	2.4	2.4	3.4	1.8	23.5	6.1	**33.8**	**17.7**	4.6	1.2	7.9	12.5
	Hornfels	6.1	4	5.5	1.5	9.1	4.6	**13.7**	**12.2**	4.6	3.7	6.1	11.9
	Marble	1.8	3.4	4.6	0.6	0.3	2.1	0.3	2.7	**26.5**	**29.9**	15.9	2.7
	Rock gypsum	0.6	0.6	4.3	2.7	3	4.3	3.7	4	**27.4**	**41.2**	13.4	2.7
	Micrite	4.3	2.1	2.7	1.2	6.4	4.6	8.5	8.8	11	5.5	**18**	**14.6**
	Shale	2.7	2.7	1.8	0.3	1.8	0.9	13.1	11.3	8.2	7.6	**24.4**	**33.8**
FD absent, blocking													
		Experiment 1: Rule-Based Categories						Experiment 1: Information-Integration Categories					
		**1a. Anthracite**	**1b. Obsidian**	**2a.** **Breccia**	**2b. Conglomerate**	**3a.** **Gabbro**	**3b. Peridotite**	**4a. ** **Basalt**	**4b.** **Hornfels**	**5a.** **Marble**	**5b. Rock gypsum**	**6a.** **Micrite**	**6b. ** **Shale**
Participants’ Responses	Anthracite	**36**	**12.4**	5.3	1.1	9.4	3.4	8.3	6	2.8	1.6	3.2	2.5
	Obsidian	**45.2**	**75.7**	6.7	0.7	1.6	0.7	1.8	2.3	0	2.1	1.6	1.4
	Breccia	0	0.9	**22.5**	**14.2**	14	10.6	2.3	5.5	5.3	0.5	2.8	3.7
	Conglomerate	0.2	0.2	**32.8**	**65.6**	8	2.5	1.4	0.9	1.1	0	1.4	1.4
	Gabbro	0.7	0.7	6.4	4.1	**12.4**	**14.2**	6.9	8.9	6.9	2.3	3.2	4.8
	Peridotite	1.8	0.9	9.6	7.3	**12.6**	**41.5**	4.6	6.4	7.6	2.5	2.1	2.5
	Basalt	4.8	1.1	2.8	0.2	21.6	10.3	**31.7**	**22.5**	5.7	2.3	7.8	13.5
	Hornfels	3.4	1.6	3.9	1.6	8.3	5.3	**14.2**	**21.1**	4.6	0.7	6	11.7
	Marble	0.7	1.8	6	0.7	1.1	1.1	0.9	1.8	**36.7**	**35.8**	10.8	0.9
	Rock gypsum	1.4	1.4	1.1	0.7	2.8	3.7	3	3.7	**18.6**	**37.2**	15.4	3.9
	Micrite	3.2	1.6	2.5	2.8	6.2	6.2	7.6	7.1	6.9	8.7	**16.5**	**9.2**
	Shale	2.5	1.6	0.5	0.9	2.1	0.5	17.4	13.8	3.9	6.4	**29.4**	**44.5**

Note. The data points showed the percentage of responses in the final classification test. For example, under the FD present, interleaving condition, when Anthracite exemplars were given, on average 57.7% of the responses were correct, whereas 30.7% of participants’ responses were Obsidian. Blue color indicates the percentage of correct response. Red color represents the percentage of near error (i.e., choosing the other rock category in the same pair).

## Appendix C

**Table A2:** The confusion matrix showcasing participants’ responses in the final classification test of Experiments 2 and 3.

FD present, interleaving													
		Experiment 2: Information-Integration Categories						Experiment 3: Information-Integration Categories					
		**4a. ** **Basalt**	**4b.** **Hornfels**	**5a.** **Marble**	**5b. Rock gypsum**	**6a.** **Micrite**	**6b. ** **Shale**	**4a. ** **Basalt**	**4b.** **Hornfels**	**5a.** **Marble**	**5b. Rock gypsum**	**6a.** **Micrite**	**6b. ** **Shale**
Participants’ Responses	Basalt	**49.4**	**22.4**	6.3	1.4	3.1	8.2	**40.7**	**22.9**	5.1	1.3	5.3	7.7
	Hornfels	**28.7**	**42**	2.6	0.9	14.8	28.4	**31.4**	**42**	2.9	1.3	10.1	30.1
	Marble	0.3	2	**51.7**	**24.1**	8	1.7	0	3.7	**54.5**	**26.1**	9.8	2.1
	Rock gypsum	0.6	2.8	**27.8**	**62.2**	9.1	2.6	1.6	3.7	**25.8**	**63.3**	8.8	3.2
	Micrite	11.4	18.5	6.3	8.2	**40.1**	**15.9**	13.3	18.6	5.6	4.8	**37.2**	**12.2**
	Shale	9.7	12.2	5.4	3.1	**25**	**43.2**	13	9	6.1	3.2	**28.7**	**44.7**
FD absent, interleaving													
		Experiment 2: Information-Integration Categories						Experiment 3: Information-Integration Categories					
		**4a. ** **Basalt**	**4b.** **Hornfels**	**5a.** **Marble**	**5b. Rock gypsum**	**6a.** **Micrite**	**6b. ** **Shale**	**4a. ** **Basalt**	**4b.** **Hornfels**	**5a.** **Marble**	**5b. Rock gypsum**	**6a.** **Micrite**	**6b. ** **Shale**
Participants’ Responses	Basalt	**48.3**	**16.3**	5.2	1	5.6	11.1	**48.4**	**17.4**	5.9	3	6.9	10.2
	Hornfels	**21.2**	**56.6**	1.7	2.1	5.6	25.3	**21.7**	**44.4**	3.9	3.6	8.6	22.7
	Marble	0.7	2.8	**51**	**25.7**	8.3	1.4	0.7	7.6	**43.4**	**25.7**	11.8	0.7
	Rock gypsum	4.2	4.9	**24**	**58**	9	2.8	2	6.9	**30.3**	**54.6**	11.2	5.3
	Micrite	8	11.5	15.3	10.1	**39.2**	**12.5**	9.2	14.8	11.8	9.9	**28.9**	**12.8**
	Shale	17.7	8	2.8	3.1	**32.3**	**46.9**	18.1	8.9	4.6	3.3	**32.6**	**48.4**
FD present, blocking													
		Experiment 2: Information-Integration Categories						Experiment 3: Information-Integration Categories					
		**4a. ** **Basalt**	**4b. ** **Hornfels**	**5a. ** **Marble**	**5b. Rock gypsum**	**6a.** **Micrite**	**6b. ** **Shale**	**4a. ** **Basalt**	**4b.** **Hornfels**	**5a.** **Marble**	**5b. Rock gypsum**	**6a.** **Micrite**	**6b. ** **Shale**
Participants’ Responses	Basalt	**46.9**	**22.2**	5.6	0.6	4.4	10	**47.9**	**23.8**	6.8	0.9	6.5	8.6
	Hornfels	**27.2**	**36.9**	3.4	5	14.4	25	**22.3**	**37.5**	2.7	4.5	15.2	25.6
	Marble	0.6	4.1	**51.2**	**28.4**	10.6	1.9	0	4.2	**51.5**	**25.6**	14.6	2.7
	Rock gypsum	3.1	8.4	**22.8**	**46.6**	14.7	5.3	3	7.1	**26.2**	**51.2**	13.1	6.8
	Micrite	12.2	18.1	9.7	12.2	**34.7**	**17.5**	12.2	17.3	10.4	11	**28.9**	**15.5**
	Shale	10	10.3	7.2	7.2	**21.3**	**40.3**	14.6	10.1	2.4	6.8	**21.7**	**40.8**
FD absent, blocking													
		Experiment 2: Information-Integration Categories						Experiment 3: Information-Integration Categories					
		**4a. ** **Basalt**	**4b.** **Hornfels**	**5a. ** **Marble**	**5b. Rock gypsum**	**6a.** **Micrite**	**6b. ** **Shale**	**4a. ** **Basalt**	**4b.** **Hornfels**	**5a.** **Marble**	**5b. Rock gypsum**	**6a.** **Micrite**	**6b. ** **Shale**
Participants’ Responses	Basalt	**47.7**	**20.3**	3.8	0.3	7.8	13.1	**47.4**	**28.5**	7.6	1.2	10.8	15.4
	Hornfels	**20.1**	**33.7**	5.5	2.6	10.2	22.1	**18.9**	**34.6**	4.9	4.4	15.1	20.1
	Marble	0.6	3.8	**54.9**	**41.9**	13.7	2.6	0	2.3	**52.9**	**38.4**	9.6	1.7
	Rock gypsum	4.1	11	**20.6**	**37.2**	17.4	10.8	6.1	8.7	**22.1**	**38.7**	15.1	8.4
	Micrite	12.2	16.9	7.3	9.3	**28.8**	**16.3**	11.6	15.4	7.8	10.8	**27**	**16.6**
	Shale	15.4	14.2	7.8	8.7	**22.1**	**35.2**	16	10.5	4.7	6.7	**22.4**	**37.8**

Note. The data points showed the percentage of responses in the final classification test. For example, under the FD present, interleaving condition, when Basalt exemplars were given, on average 49.4% of the responses were correct, whereas 28.7% of participants’ responses were Hornfels. Blue color indicates the percentage of correct response. Red color represents the percentage of near error (i.e., choosing the other rock category in the same pair).
